# Novel Deposition Method of Crosslinked Polyethylene Thin Film for Low-Refractive-Index Mid-Infrared Optical Coatings

**DOI:** 10.3390/s23249810

**Published:** 2023-12-14

**Authors:** Taeyoon Jeon, Jieun Myung, Changsoon Choi, Komron Shayegan, Scott M. Lewis, Axel Scherer

**Affiliations:** Applied Physics and Materials Science, California Institute of Technology, 1200 East California Boulevard, MC 200-79, Pasadena, CA 91125, USA

**Keywords:** mid-infrared optical coating, polyethylene, crosslinking, low-refractive-index materials

## Abstract

Mid-infrared optics require optical coatings composed of high- and low-refractive-index dielectric layers for the design of optical mirrors, filters, and anti-reflection coatings. However, there are not many technologies for depositing a material with a refractive index of less than 2 and a low loss in the mid-infrared region. Here, we present a unique deposition method of crosslinked polyethylene thin film for mid-IR optical filter design. Polyethylene has a refractive index of 1.52 in the mid-infrared region and a small number of absorption peaks, so it is useful for making optical filters in the mid-infrared region. Only 1 keV of energy is required to crosslink the entire film by irradiating an electron beam while depositing polyethylene. In addition, crosslinked polyethylene thin film has high mechanical strength, so there is no cracking or peeling when used with germanium. This allows for the use of crosslinked polyethylene as a low refractive index for mid-infrared optical coating.

## 1. Introduction

The mid-infrared (mid-IR) region is a molecular fingerprint region which is widely used for molecular detection [1]. In this mid-IR region, various optical devices such as Fourier transform infrared spectroscopy (FTIR) [2] and quantum cascade laser (QCL) [3] are used for molecular detection. In addition, there have been studies to miniaturize molecular sensors in the mid-infrared region using resonators such as the Fabry–Perot filter [4], frequency comb [5,6], and metasurface [7,8]. One of the major limitations in designing mid-IR optical systems is that there are few low-refractive-index materials with low absorption in the mid-IR region. The commonly used high-refractive-index materials in the mid-infrared region are germanium and silicon [9], and the refractive indices of each material are about 4 and 3.4, respectively [10,11]. However, low-refractive-index materials used in the mid-infrared region, such as zinc sulfide (ZnS) [12] and zinc selenide (ZnSe) [13], have a refractive index greater than 2 in the mid-infrared region, making it difficult to maximize the difference in refractive index when making an optical device. This results in a narrow bandwidth of the optical filter, and many layers need to be deposited to improve the refractive index contrast in the optical filter design [14]. Recently, studies on chalcogenide glasses have been extensively researched to find other infrared transparent alternative materials due to its transparency in the infrared region. However, these materials are not easy to prepare, and the refractive index of the chalcogenide glasses is also over 2 [15,16]. A fluoride material such as calcium fluoride (CaF_2_) can be used as a substrate for a low-refractive-index material [17]. However, in order to make an optical filter, a technique for depositing as a thin film is required. In addition, since most fluoride materials such as CaF_2_, yttrium fluoride (YF_3_), and cerium fluoride (CeF_3_) begin to absorb light over 10 μm, there is a limit to use them over 10 μm region [18].

Here, we propose the use of polyethylene (PE) as a low-refractive-index material in the mid-IR region because PE has low absorption in the mid-infrared region and a refractive index of around 1.53 [19]. However, in order to manufacture an optical filter, it is essential to deposit a robust PE thin film. There have been studies on the preparation of a polyethylene thin film using a solution process and thermal evaporation by using a vacuum chamber. However, the solution process (spin-coating) has the problem that the sample and solution must be heated at a high temperature (180 °C) to dissolve the PE due to the low solubility of PE in the solvent at room temperature, and the resulting film has some voids in the film [20]. This is not sufficient for optical filter design. In addition, thermal evaporation of the polymer usually decomposes the polymer during heating, resulting in low mechanical stability of the deposited film [21]. Research on crosslinking in polyethylene using electron beam irradiation to enhance the mechanical stability of polyethylene film has been conducted [22,23]. Here, we incorporated electron beam irradiation into the process of evaporating polyethylene via thermal evaporation, aiming to achieve both highly controllable thickness of smooth polyethylene film and improve the mechanical stability of the deposited film.

**Figure 1:**
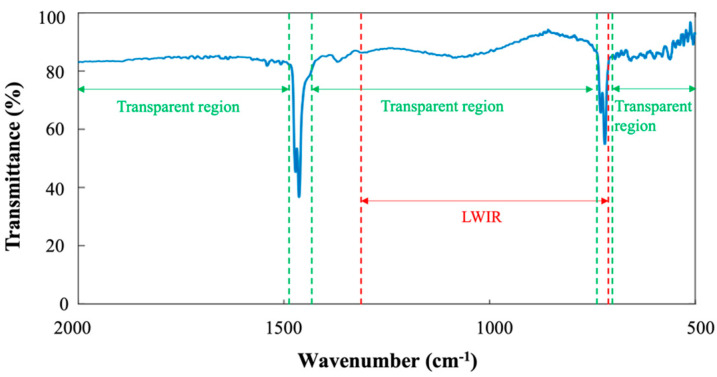
Transmittance spectrum of PE in the mid-IR region. The absorption peaks are at 1466 cm^−1^ and 723 cm^−1^. The transparent region of PE in the mid-IR is indicated by green arrows, and the LWIR region is indicated by red arrows.

We developed the thin film deposition method of crosslinked polyethylene (XPE) by using an electron beam-assisted thermal evaporation system. By irradiating electrons while depositing PE, it is possible to deposit XPE thin film that is mechanically stable and has a low refractive index in the mid-infrared range. Polyethylene is one of the polymers with the fewest number of absorption peaks in the mid-infrared region (Figure 1) because of its simple chemical structure [24]. In the mid-IR range (from 5 μm to 20 μm or from 2000 cm^−1^ to 500 cm^−1^), PE has an absorption peak of 1466 cm^−1^ (6.82 μm) and 723 cm^−1^ (13.83 μm). The peak at 1466 cm^−1^ is C–H deformation vibrations in –(CH_2_)_n_–, and the peak at 723 cm^−1^ is from C–C rocking vibrations in –(CH_2_)_n_– [25]. These peak positions have little overlap in the long-wave infrared (LWIR) range, which is 7.5–14 μm (wavelength scale) or 1333.3 cm^−1^ to 714.3 cm^−1^ (wavenumber scale). This LWIR range is the area outside the absorption peaks of moisture in the air, and it is the bandpass filter transmittance range used in mid-IR optical devices such as thermal imaging cameras [26]. Therefore, when polyethylene is used in this LWIR region, the absorption peaks of polyethylene hardly overlap with the LWIR region, so it is possible to manufacture an optical device with polyethylene in this region. The refractive index of PE in the mid-infrared region is around 1.53, which is much smaller than the conventional low-refractive-index materials (ZnS and ZnSe). The extinction coefficient value of the PE film in the transparent region is on the order of 10^−3^, and at the peak position, the extinction coefficient value is on the order of 10^−2^ [19].

## 2. Deposition Method of XPE Thin Film

**Figure 2:**
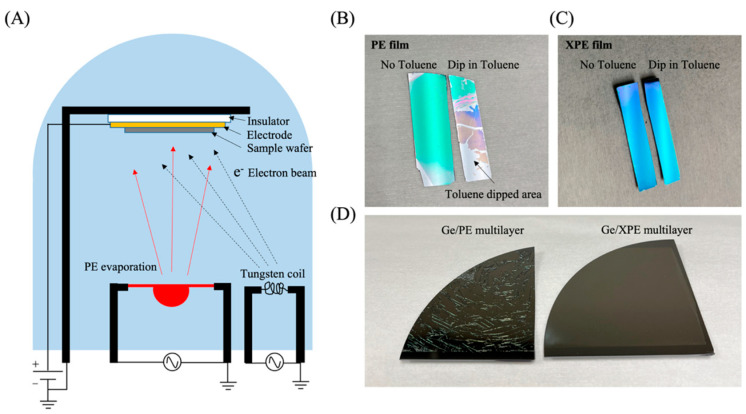
(**A**) Schematic showing a vacuum chamber for depositing crosslinked polyethylene thin films. Electrons generated in the tungsten coil are accelerated to the sample wafer due to the electric potential. Polyethylene monomers are crosslinked as soon as they are deposited on the sample by accelerated electrons. (**B**) Digital camera image of PE thin film. On the left is a PE thin film on a silicon wafer that has not been immersed in toluene. The image on the right is after immersion in toluene. (**C**) Digital camera image of the XPE thin film. On the left is a PE thin film on a silicon wafer that has not been immersed in toluene. The image on the right is after immersion in toluene. (**D**) Digital camera image when Ge was deposited on PE (left) and XPE (right). When Ge is deposited on PE, cracks occur, whereas, in the case of XPE, there is no crack on the film.

The XPE thin film deposition system is designed as shown in Figure 2A. PE molecules are heated in a tungsten boat, evaporated, and deposited on the sample substrate. We purchased polyethylene raw material from Sigma-Aldrich, and its molecular weight is about 4000. To evaporate the polyethylene, the boat temperature should be increased up to 350 °C. The deposition rate is 1 nm/s. An accelerated electron beam is irradiated to the substrate for the crosslinking of the deposited PE molecules. An electron beam is generated in a tungsten coil heated via an applied current. This thermionic emission enables electron emission by adding thermal energy to the electron to overcome the work function of the tungsten [27]. Then, generated electrons are accelerated by an electric potential (1 kV). A sample wafer is placed on the surface of a positively charged electrode, and an insulator is placed between the electrode and the sample holder, which can accelerate the electron beam to the sample surface. With this technique, PE can be crosslinked at the same time as PE molecules are deposited. In order to check whether the deposited PE film is crosslinked, the PE film deposited on the silicon wafer was immersed in a heated toluene solution. The PE film deposited without e-beam radiation is soluble in toluene, as shown in Figure 2B, but PE film deposited with e-beam radiation is not soluble in toluene, as shown in Figure 2C, due to crosslinking of PE. To measure the crosslinking density more precisely, the thickness of crosslinked PE film was measured using an interferometry method, and the thickness was checked after dipping in the toluene for 30 min. The interferometry measurement showed the same thickness of film after dipping it ©n the toluene, indicating that it did not dissolve and polyethylene film completely transformed into a gel form (Figure S1). This crosslinking can also increase the mechanical properties of film. By depositing a thin film of Ge on uncross-linked PE and XPE films, the mechanical properties of the films can be checked. As can be seen in Figure 2D, the deposition of germanium on an uncross-linked PE film causes a lot of cracking in the film. This is because the uncross-linked PE film cannot withstand the internal stress of the film. On the other hand, when Ge is deposited on the XPE film, cracks do not occur in the film.

**Figure 3:**
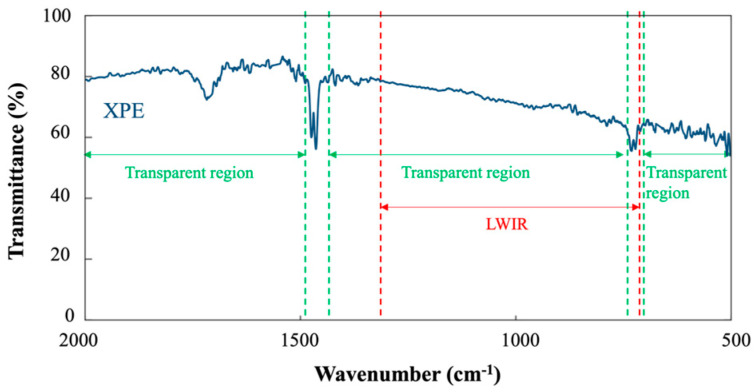
Transmittance spectrum of XPE in the mid-IR region shows an absorption peak around 1720 cm^−1^ area. This peak corresponds to the C=O stretch bond, and it is attributed to the outgassing of oxygen during the evaporation. The XPE remains transparent in the LWIR region.

The transmittance of the XPE thin film is shown in Figure 3. The spectrum exhibits an absorption peak at 1720 cm^−1^ area, which corresponds to the C=O stretch bond. This is due to the outgassing of oxygen during the thermal evaporation of polyethylene inside the vacuum chamber and can be solved by performing a degassing process before deposition. The energy dispersive x-ray spectroscopy (EDX) measurements for both the raw material and evaporated PE film are presented in Figure S2. The results indicate that the raw material is 100% carbon, while the evaporated XPE contains oxygen. Nevertheless, the XPE remains transparent in the LWIR region.

## 3. Monte Carlo Simulation

**Figure 4:**
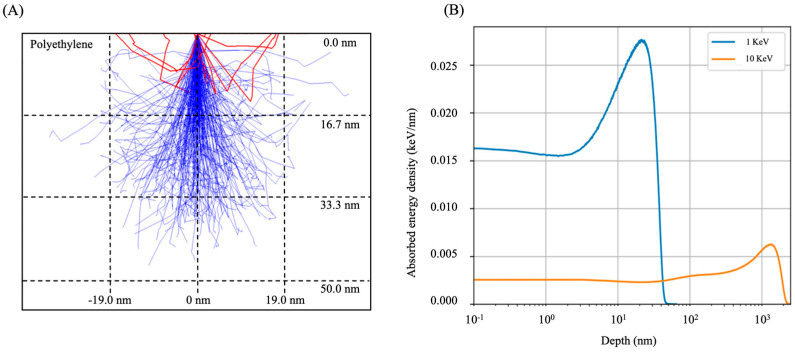
(**A**) Monte-Carlo simulation of trajectories of 200 electrons with 1 keV energy in a polyethylene layer. Blue trajectories show absorbed electrons, while red ones indicate backscattered electrons. (**B**) Absorbed energy density at each depth in a polyethylene layer calculated by using 100,000 number of electrons. The absorbed energy density curves have their maxima at 21.1 nm for 1 keV electrons and 1.35 μm for 10 keV electrons, respectively.

The electron beam penetration depth can be calculated using the CASINO Monte-Carlo program [28]. The 200-electron trajectory with 1 keV energy inside the PE film is shown in Figure 4A. The blue trajectories show absorbed energy inside of PE film, while the red trajectory shows backscattered electrons. The probability of backscattering is about 20%. To calculate the absorbed energy distribution according to the PE polymer depth, 100,000 electrons with 1 keV are irradiated to the PE film in the simulation. The absorbed energy density along the penetration depth from the average of 100,000 electrons’ trajectory is shown in Figure 4B. The integration of all data along the depth of the PE film is about 0.8 keV, meaning that 80% of the irradiated electrons are absorbed, and 20% are backscattered. The peak position of the electron absorption energy density is around 21.1 nm in depth, and the absorption energy is almost zero in the 50 nm depth in the case of 1 keV electrons. Since the deposition rate of PE molecules in our system is 1 nm/s and PE molecules are exposed to an electron beam as soon as they are deposited on the substrate, this 1 keV is sufficient to crosslink the film. This is a huge advantage in terms of the required electron beam energy and crosslinking uniformity of the film. For example, irradiating an electron beam after finishing PE film deposition requires 10 keV to penetrate a 1 μm thick PE polymer, as shown orange curve in Figure 4B, and this required energy will be much bigger for deeper penetration. Compared to this energy, a system with simultaneous deposition and irradiation requires much less electron energy because PE deposition and crosslinking are performed simultaneously. Also, this results in uniform crosslinking density along the film depth.

## 4. Mid-IR Optical Filter Fabrication

**Figure 5:**
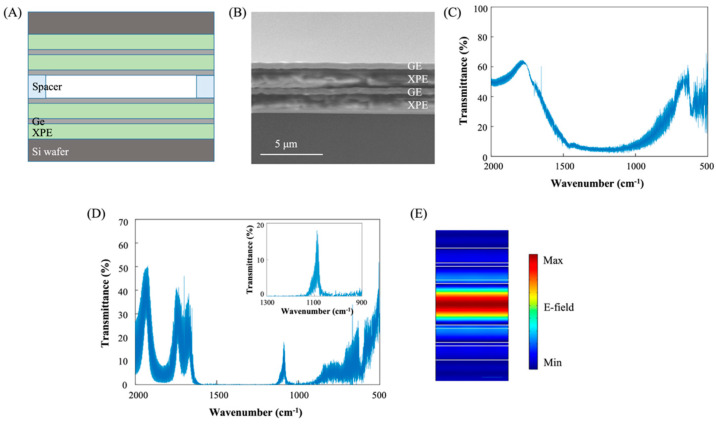
(**A**) Schematic diagram showing the design of a mid-IR optical filter composed of two DBRs made of Ge and XPE. (**B**) SEM image of a DBR consisting of two pairs of Ge and XPE layers. (**C**) Transmission spectrum of the Ge/XPE mirror in (**B**). (**D**) Transmission spectra of a Ge/XPE filter when two DBRs are combined with an air cavity in between. Inset is a magnified spectrum at transmittance peak position. (**E**) Calculated electric field intensity distribution inside the cavity when resonant wavelength light passes through the filter.

A mid-IR optical filter can be fabricated using XPE thin film deposition technology. The overall design of the fabricated optical filter is shown in Figure 5A. The optical filter has a structure in which two Distributed Bragg reflectors (DBR) face each other with a spacer thickness apart. The spacer thickness determines the cavity thickness of the filter and the resonance frequency of the filter. The DBR is composed of Ge (as a high-refractive-index material) and XPE (as a low-refractive-index material). The target wavelength of DBR is 9 μm, so the thickness of each layer is 9/4n, where *n* is the refractive index of each layer. So, the thickness of Ge is about 560 nm, and XPE is about 1480 nm. A cross-sectional view of one deposited DBR is shown in Figure 5B, and the transmittance spectrum of one DBR is shown in Figure 5C. The bandwidth of DBR was designed to target the LWIR range. To create a Fabry–Perot filter, two DBRs are combined, creating a cavity between them. For an optical filter, a thin film of polydimethylsiloxane (PDMS) is placed between the two DBRs to form an air cavity. The transmittance spectrum of the optical filter is shown in Figure 5D. The resonance peak is at 1085 cm^−1^ (9.2 μm). The resonant wavelength of light is enhanced in the cavity region (Figure 5E). This filter has a huge advantage over other low-index materials as it can produce sharp transmission peaks with only four pairs of layers due to the large refractive index contrast.

## 5. Discussion

Because we developed a thin film deposition method for robust polyethylene suitable for mid-IR optics, it can be utilized as a low-refractive-index material in mid-IR optical films. When compared to conventional mid-IR low-refractive-index materials, polyethylene offers distinct advantages.

One advantage is its low refractive index. In comparison to other commonly used low-refractive-index materials in the mid-IR region, such as ZnS and ZnSe, which have refractive indices over 2, polyethylene possesses a refractive index of about 1.53 in the mid-IR region.

The second advantage is that polyethylene is transparent at wavelengths exceeding 15 μm. This is a significant benefit when contrasted with other fluoride-based materials like CaF_2_, as many fluoride materials begin to absorb light beyond 12 and 13 μm. Therefore, if a refractive index lower than 2 is required over the 15 μm wavelength region, polyethylene can be an ideal material. The refractive indices of conventional low-refractive-index materials are compared with polyethylene, along with their transparent regions, in Table 1.

This low refractive index gives XPE a significant advantage in terms of wide bandwidth when employed in DBR designs. Furthermore, fewer layers are needed to achieve low transmittance (or high reflectance) for the mirror.

**Figure 6:**
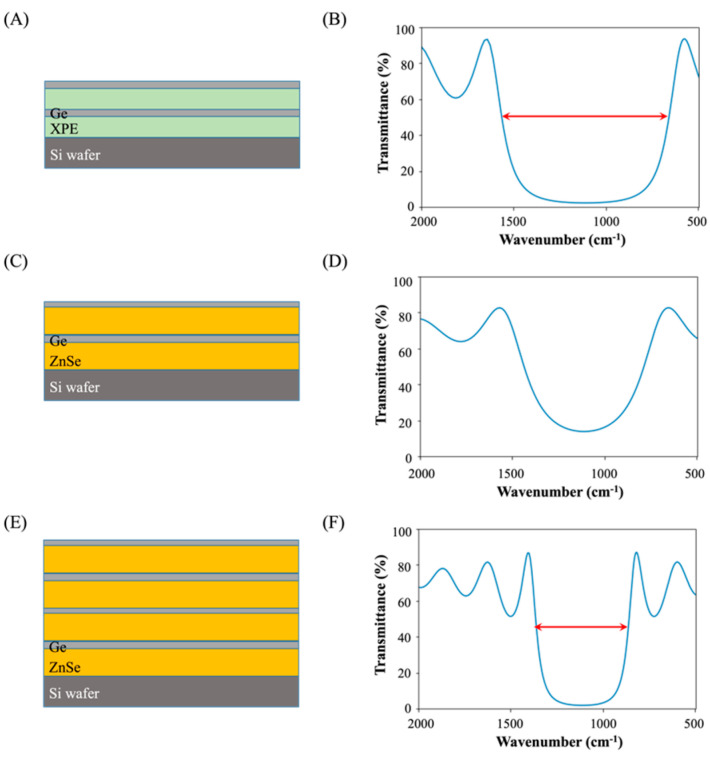
(**A**,**C**,**E**) Schematic diagrams of two pairs of Ge/XPE DBR (**A**), two pairs of Ge/ZnSe (**C**), and four pairs of Ge/ZnSe (**E**). (**B**,**D**,**F**) Calculated transmittance spectra of two pairs of Ge/XPE DBR (**B**), two pairs of Ge/ZnSe (**D**), and four pairs of Ge/ZnSe (**F**). Red arrows in (**B**,**F**) indicate the bandwidth of the free spectral range.

To verify this effect, the transmission spectra of DBR using two different low-index materials, XPE and ZnSe, are calculated. The target wavelength of DBR is 9 μm. The thickness of each layer is determined by 9/4 *n*, where *n* is the refractive index of the respective material. For germanium (Ge), *n* is 4; for XPE, it is 1.53; and for ZnSe, it is 2.4. As shown in Figure 6A,B, the bandwidth at the half-maximum of transmittance spans from 657 cm^−1^ (15.2 μm) to 1565 cm^−1^ (6.4 μm) for the two pairs of Ge/XPE. The transmittance value at the center wavelength (9 μm) is 2.4%. However, the center wavelength transmittance for the two pairs of Ge/ZnSe is 13.9% (Figure 6C,D), which is substantially higher than the two pairs of Ge/XPE.

To achieve a transmittance comparable to two pairs of Ge/XPE (about 2%), four pairs of Ge/ZnSe are required, as demonstrated in Figure 6E,F. The bandwidth at the half-maximum of transmittance for these four pairs of Ge/ZnSe ranges from 866 cm^−1^ (11.5 μm) to 1356 cm^−1^ (7.4 μm), and transmittance at the center wavelength is 1.9%. Therefore, by using XPE for low-refractive-index materials instead of using ZnSe, the number of multilayers needed to achieve approximately 98% reflectance at the center wavelength is halved, and the free spectral range is considerably expanded.

## 6. Conclusions

This first result using XPE as mid-IR optics shows that polymers can be used to design optical filters in the mid-IR region. Electron beam irradiation during the evaporation of PE molecules allowed the preparation of crosslinked PE thin films with high mechanical stability and high smoothness. Because the e-beam irradiates the PE molecule as it deposits onto the substrate, the resulting crosslinking density can be uniform throughout the entire film. And it has been shown that this XPE film deposition technique can be used to fabricate a mid-IR optical filter. Compared to other low-index materials used in mid-infrared optics, the refractive index of XPE is much lower, so the bandwidth is wider, and fewer layers are required to design high-Q filters. This technique shows the potential for use with other polymers for mid-IR optics by avoiding the absorption regions of the polymer. Additionally, the new polymer thin film deposition technique (electron beam radiation during the physical vapor deposition) can be used in areas where robust thin polymer deposition is needed, like biological applications [29] and electronic applications using polymer [30,31].

## Figures and Tables

**Table 1:** Comparison of refractive index and transparent region between polyethylene and conventional low-refractive-index materials at mid-IR region. The refractive index values of ZnS, ZnSe, Chalcogenide glass, and CaF_2_ are sourced from references [12,13,16,17] respectively.

Materials	Refractive Index at 10 µm	Transparent on between 5 µm to 20 µm
Polyethylene	1.53	Transparent up to 20 µm, except for two peaks positioned near 6.8 µm and 13.8 µm
Chalcogenide Glass	Over 2.5 (change depending on the composition)	Transparent up to 20 µm
ZnS	2.2	Transparent up to 14 µm
ZnSe	2.4	Transparent up to 14 µm and transmittance gradually decrease up to 20 µm
CaF_2_	1.3	Transparent up to 8 µm and transmittance gradually decrease and become zero at about 12 µm

## Data Availability

Data are contained within the article.

## Supplementary Materials

The following supporting information can be downloaded at https://www.mdpi.com/article/10.3390/s23249810/s1. Figure S1: Thickness measurement of XPE film before and after dipping in a toluene; Figure S2: EDX measurement data of raw polyethylene powder and evaporated XPE thin film.
