# Supplementary material for: Novel Deposition Method of Crosslinked Polyethylene Thin Film for Low-Refractive-Index Mid-Infrared Optical Coatings
Source: Sensors (Basel). 2023 Dec 14;23(24):9810. doi: 10.3390/s23249810 (PMC10748295; doi:10.3390/s23249810)
Supplement: Supplementary file 1 [file sensors-23-09810-s001.zip › sensors-2676309-supplementary.pdf]

Supporting Information

Novel Deposition Method of Crosslinked Polyethylene Thin Film for Low-Refractive-Index Mid-Infrared Optical Coatings

Taeyoon Jeon, Jieun Myung, Changsoon Choi, Komron Shayegan, Scott Lewis, and Axel Scherer

Applied Physics and Materials Science, California Institute of Technology, 1200 East California Boulevard, MC 200-79, Pasadena, California, 91125, USA

1. Thickness measurement of XPE film before and after dipping in a toluene

To check the crosslinking density and uniformity across the film, the XPE thin film was immersed in toluene, and its thickness variation was measured. Even after immersion in toluene for 30 minutes, the thickness of the XPE thin film did not change. This means that the film is thoroughly and uniformly crosslinked throughout its entire structure. This is a significant advantage compared to the conventional e-beam radiation method for crosslinking the polymers, as the conventional approach tends to create higher crosslinking density in the top layer while leaving the lower area without sufficient crosslinking.

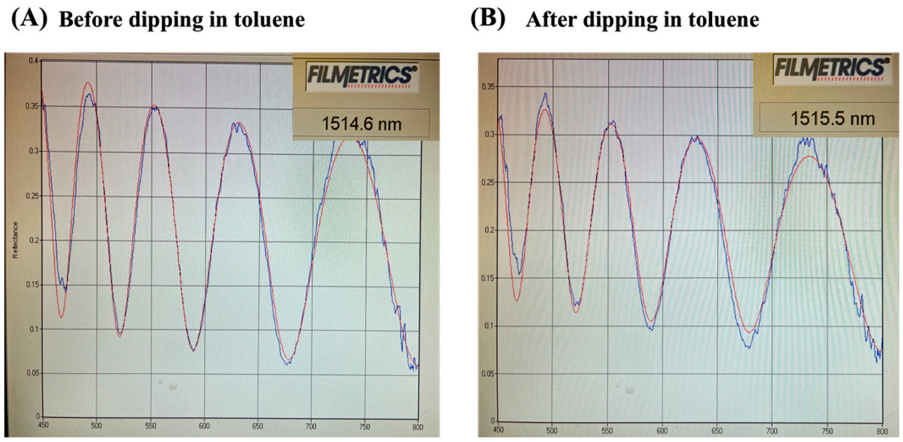

Figure S1.
(A-B) Interferometry measurement of XPE film thickness before dipping in toluene (A) and after dipping in toluene (B).

2. EDX measurement data of raw polyethylene powder and evaporated XPE thin film

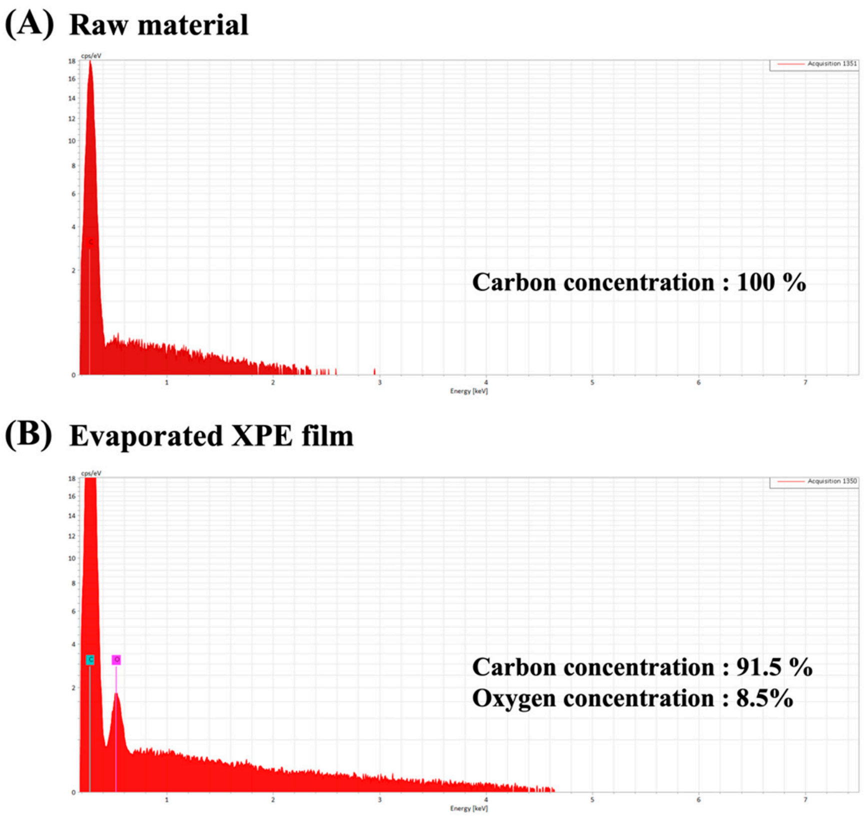

Figure S2.
(A) EDX measurement of raw polyethylene power. It shows 100% carbon. (B) EDX measurement of evaporated XPE thin film. The composition is 91.5 % carbon and 8.5 % oxygen. Oxygen comes from the outgassing during thermal evaporation.
